# Analysis of the Behavior of Deep Eutectic Solvents upon Addition of Water: Its Effects over a Catalytic Reaction

**DOI:** 10.3390/molecules29143296

**Published:** 2024-07-12

**Authors:** Paola R. Campodónico, Jazmín Alarcón-Espósito, Jackson J. Alcázar, Belén Olivares, Cristian Suárez-Rozas

**Affiliations:** 1 Centro de Química Médica, Instituto de Ciencias e Innovación en Medicina, Facultad de Medicina, Clínica Alemana Universidad del Desarrollo, Santiago 10021, Chile; jackson.alcazar@udd.cl (J.J.A.); molivares@udd.cl (B.O.); cristiansuarez@udd.cl (C.S.-R.); 2 Helen and Robert Appel Alzheimer’s Disease Research Institute, Brain and Mind Research Institute, Weill Cornell Medicine, New York, NY 10021, USA; jfa4002@med.cornell.edu

**Keywords:** deep eutectic solvents, lipases, biocatalysis, solvent effect, hydrogen bonds

## Abstract

This study presents the potential role of deep eutectic solvents (DESs) in a lipase-catalyzed hydrolysis reaction as a co-solvent in an aqueous solution given by a phosphate buffer. Ammonium salts, such as choline chloride, were paired with hydrogen bond donors, such as urea, 1,2,3-propanetriol, and 1,2 propanediol. The hydrolysis of *p-*nitrophenyl laureate was carried out with the lipase *Candida antarctica* Lipase B (CALB) as a reaction model to evaluate the solvent effect and tested in different DES/buffer phosphate mixtures at different % w/w. The results showed that two mixtures of different DES at 25 % w/w were the most promising solvents, as this percentage enhanced the activities of CALB, as evidenced by its higher catalytic efficiency ($\frac{k_{cat}}{K_{M}}$). The solvent analysis shows that the enzymatic reaction requires a reaction media rich in water molecules to enable hydrogen-bond formation from the reaction media toward the enzymatic reaction, suggesting a better interaction between the substrate and the enzyme-active site. This interaction could be attributed to high degrees of freedom influencing the enzyme conformation given by the reaction media, suggesting that CALB acquires a more restrictive structure in the presence of DES or the stabilized network given by the hydrogen bond from water molecules in the mixture improves the enzymatic activity, conferring conformational stability by solvent effects. This study offers a promising approach for applications and further perspectives on genuinely green industrial solvents.

## 1. Introduction

Integrated knowledge based on experiments, theory, and computational tools is fundamental in order to elucidate a complete picture of enzyme catalysis [1]. The first model of enzyme catalysis was “lock and key”, which proposed that the binding of a substrate molecule (S) to activate the site on the enzyme (E) results in the activation of the substrate, with a reactive conformation between the substrate and the enzyme complex (ES) [2]. A modified version, based on ground-state destabilization, showed the “key does not require fitting the lock perfectly; however, it exerts strain on it”. [3]. Transition state (TS) theory hypothesized a preferential binding between enzyme–TS over the substrate or product (P), which results in an increased stabilization (lower free energy) along the reaction coordinate [4] given by electrostatic, steric, or hydrogen bonding (HB) or solvation effects between the substrate and the active site cavity of the enzyme [5].

**Scheme 1:**
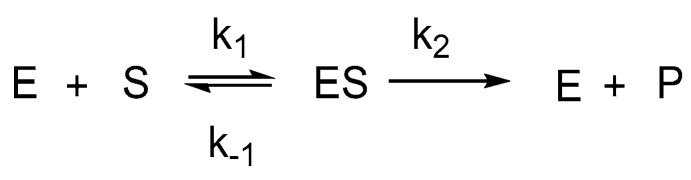
General picture of Michaelis–Menten mechanism.

Michaelis–Menten’s description for enzymatic reactions has been broadly used in order to contrast useful information about kinetic data for the enzymatic reaction performed in aqueous media and other reaction media [6,7]. These kinetics data have had an essential role in the characterization of the enzyme activity [6]. Scheme 1 shows the Michaelis–Menten mechanism, in which a substrate binds reversibly to the enzyme to form an ES complex. This complex proceeds to P, and E is regenerated for the next catalytic cycle. Considering the formation of product ($k_{2}$) as the slow step, $\left[E_{T}\right]=\left[E\right]+\left[\mathrm{ES}\right]$ is the total enzyme concentration, and $v_{0}={\;k}_{2}\left[\mathrm{ES}\right]$. The Michaelis–Menten equation gives a hyperbolic dependence of the enzyme velocity (v) with the substrate concentration $\left[S\right]$, where the Michaelis constant ($K_{M}$) or substrate affinity constant is $K_{M}=\frac{\left(k_{-1}+k_{2}\right)}{k_{1}}$. And, $v_{\max}$ is the maximum enzyme velocity ($v_{\max}={\;k}_{2}\left[E_{T}\right]$) achieved by the catalytic reaction at saturating $\left[S\right]$. Then, the Michaelis–Menten equation is $v_{0}=\frac{v_{\max}\left[S\right]}{K_{M}+\left[S\right]}$. The catalytic efficiency $\left(\frac{k_{\mathrm{cat}}}{K_{M}}\right)$ is defined by how efficiently an enzyme converts an S into P, when $k_{2}$ = $k_{\mathrm{cat}}$ = $\frac{v_{\max}}{\left[E_{T}\right]}$. Therefore, the catalytic efficiency is $\frac{\left(k_{2}\;k_{1}\right)}{\left(k_{-1}+k_{2}\right)}$ [8].

Since the 1990s, studies of enzymes in non-aqueous media have been reviewed, indicating that they can be useful reaction media with an impact on green chemistry (CG) [9,10]. GC emphasizes the design of chemical products and processes with the aim of strongly reducing, or eliminating, chemicals that may become hazardous when transferred to the environment as waste [11,12]. These studies have mainly considered proteases and lipases because proteases need to retain a critical level of hydration in the non-aqueous environment in order to maintain their enzymatic activities [13]. *Candida antarctica* Lipase B (CALB) is a suitable enzyme model for analyzing solvent effects because it is a very stable enzyme compared to other lipases [14]. Results based on the rate of the enzymatic reaction highlight the key role of the hydrophobicity and polarity of the environment in maintaining this first shell of the solvent [15]. Studies on the solvent effect on enzymatic catalysis are complex because they must consider (i) differences in enzyme hydration [16] and (ii) solvation of enzyme and substrate, respectively. The key role of the solvent effects is centered over the enzymatic activity in each studied solvent, [14,17] which is a mainly unstudied open area to be explored.

Recent advancements in enzyme-compatible sustainable solvents have brought attention to deep eutectic solvents (DES). These DESs are substances formed by the combination of at least two compounds, a hydrogen acceptor (HBA) and a hydrogen-bond donor (HBD), which are typically solid at room temperature. These raw materials, combined in a given molar ratio, lead to the formation of a mixture that exhibits a considerably lower melting point than its original constituents, becoming liquids at room temperature [18,19] and exhibiting a much lower melting point at the eutectic than what is expected for the ideal behavior of the liquid mixture [20]. This simple preparation is 100% atom economic, and it does not require further purification. Thus, catalysis in natural deep eutectic solvents (NADES) is a completely green chemical process. Then, NADES can be prepared from primary metabolites, such as sugars, amino acids, and organic acids [21]. DES shows a high combinatorial flexibility resulting from the high number of HBD and HBA species that enable the tuning of chemical properties. On the other hand, recently, there has been reported that the variation of the molar ratio between the two DES components allows the mentioned combinatorial flexibility [22]. Moreover, they are cheaper to prepare because their preparation is based on low-cost materials with easy preparation and purification methods [23,24]. DES offers several advantages from the perspective of GC and the solvent effects, such as (i) environmental friendliness, because their components are biodegradable and generally of low environmental risk, and (ii) low toxicity, compared to many conventional solvents (CS) that tend to be less toxic and safer for various applications. However, the toxicological and biodegradability information is still scarce [25,26]. Pretti et al. has denoted that a natural system can be influenced by biotic and abiotic factors and may synergistically act in the presence of DES or NADES [26]. (iii) Other effects are low volatility, as they tend to have higher boiling points than CS [27], and (iv) adaptability and selectivity, as they can be designed to suit numerous and specific needs (tunable).

**Scheme 2:**
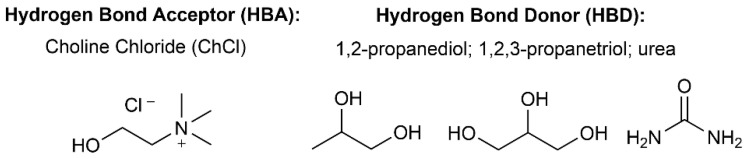
Structures of HBA and HBD of DES used in this study.

Nian et al. [28] reported that CALB is activated and stabilized by an HB interaction established in betaine-glycerin and choline chloride-glycerol in comparison to ethanol while CALB in betaine-xylilol and choline chloride-xylilol decreases. In this study, the HBA was choline chloride (ChCl) and the HBDs were urea, 1,2,3-propanetriol (glycerol), and 1,2-propanediol (see Scheme 2 below). Note that the acronyms used in this study include CU (choline-urea), CG (choline-1,2,3-propanetriol), and CE (choline-1,2-propanediol), respectively.

Many primary metabolites can form NADES, suggesting that formation may play a key role in biological functions [29]. The pioneer work of Gill et al. showed that enzymes are able to retain their activities in NADES, improving it in comparison to conventional organic solvents (COS) [30]. Improved activities of lipases in DES based on choline chloride-urea were reported by Kazlauskas et al. [31] Note that urea is a potent protein denaturant through the disruption of intramolecular HB. However, in CU, the moieties choline (Ch) and Cl^−^ form HB with urea, preventing its diffusion into the protein core and increasing the enzyme stability [29]. Zhao et al. introduced the lipase-compatible DES based on ChCl-glycine and choline acetate-glycine in 1:2 and 1:1.5 molar ratios, respectively [32].

On the other hand, water added in some DESs improved the enzyme activity and also inhibited the side reactions [33]. Thus, the enzymatic reactions can be carried out in different reaction media and, finally, in non-conventional reaction media, such as ionic liquids (IL) and DES [14,17,34,35]. The selection of an appropriate solvent can drastically affect the course of a chemical transformation because it can be determined by the ability of the solvent to interact with the solute, enzyme, and TS structures along the reaction pathway. This problem will be resolved with experimental information and by considering the main factors that affect enzymatic reaction, including HB basicity/acidity, polarity, viscosity, and solvent network [14]. Importantly, mixtures of reaction media have been studied very little, thus opening a promising approach for applications and further perspectives for uses as genuinely green industrial solvents. This study evaluated the performance of CALB in buffer phosphate (pH = 7) as a reference solvent and mixtures of DES/buffer phosphate at different %w/w in order to evaluate how the solvent composition exerts an effect on the biocatalysis. The investigated reaction corresponds to the hydrolysis of the substrate *p-*nitrophenyl laurate (*p-*NPL) in each reaction media (see Scheme S1 in the Supplementary Material, SM). Shao et al. reported that DES molecules form a solvation shell around the enzyme, conferring flexibility and a specific structure. In addition, water molecules would change this network, affecting the conformation and active site of the enzyme [36]. Maugeri et al. performed whole-cell biocatalysis in choline acetate-glycerol, demonstrating that, in 20 vol% water, the biocatalysis was stabilized [37]. In this way, aqueous deep eutectic solvent solutions emerge as a promising reaction media applied to catalysis [38,39,40,41]. However, other authors consider water as an impurity [42]. Edler et al. opened a significant debate about the amount of water tolerated by each system and the effect of the transition water over water-in-DES systems to DES-in-to-aqueous solutions of DES components [43]. Then, the role of water must be analyzed considering its effect on the physico-chemical properties and its effect over the DES components due to the water molecules strongly interacts with DES structure. To resolve this controversy, this study considers the mentioned DESs (see Scheme 1) and its mixtures in a broad range of % w/w that were analyzed across physicochemical parameters, including conductivity, density, and solvatochromic solvents parameters. All of these factors play a role in determining (i) activity/selectivity of the enzyme in each reaction media; (ii) nature of the solvent and its relationships with the enzyme activity; (iii) interactions between solvent and enzyme; (iv) bond site between solvent and enzyme; and (v) HB interactions.

## 2. Results and Discussion

**Figure 1:**
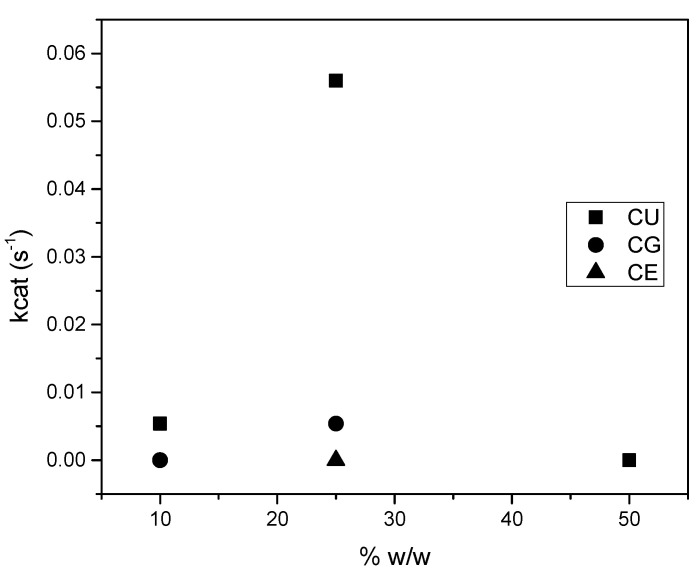
Relationship between $k_{\mathrm{cat}}$ vs. % w/w in each solvent mixture for all the studied DES.

**Figure 2:**
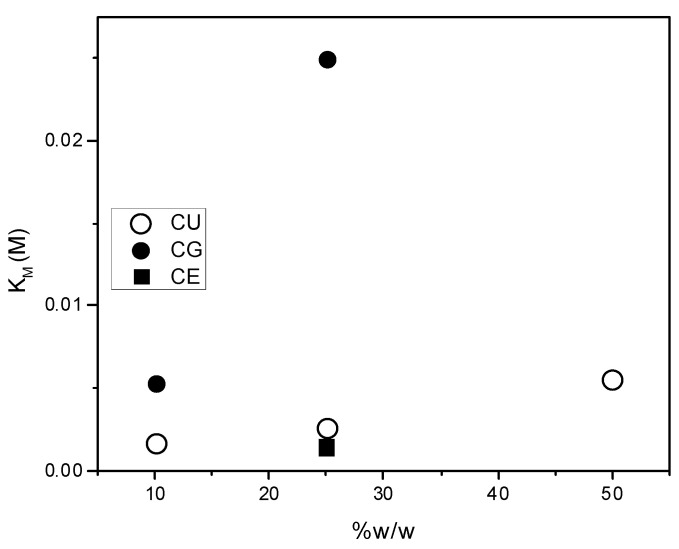
Relationship between $K_{M}$ vs. % w/w in each solvent mixture studied.

**Figure 3:**
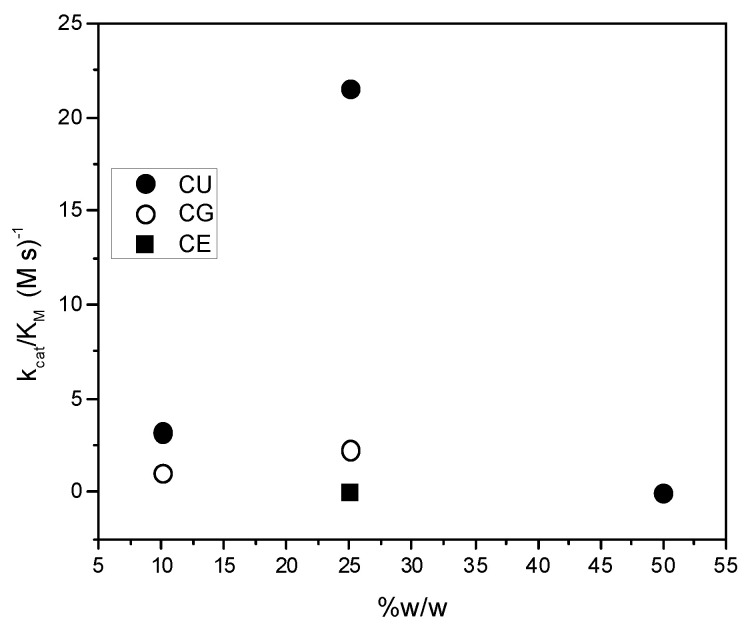
Relationship between $\left(\frac{k_{\mathrm{cat}}}{K_{M}}\right)$ vs. % w/w in each solvent mixture studied.

This study considered the kinetic analysis of the hydrolysis reaction of p-NPL in the presence of CALB in three-deep eutectic solvents, namely CU, CG, and CE, at room temperature and their DES/buffer mixtures in a broad range of %w/w (more details in Scheme S1 in SM). Note that only some mixtures allowed for performing the kinetic study, which was attributed a priori to viscosity and solubility effects. Mixtures at 10, 25, and 50% w/w were kinetically evaluated. The Lineweaver–Burk relationships analyzed in each one of those solvent mixtures obtained the catalytic parameters ($K_{M}$ and $v_{\max}$), which are used in order to explore the kinetic behavior [14,17] (more details in Tables S1–S3 and Figures S1–S6 in SM). Note that, in all the Figures, the DES amount increases in the same order of % w/w. Figure 1 shows the relationship between $k_{\mathrm{cat}}$ vs. %w/w in each studied solvent mixture. The $k_{\mathrm{cat}}$ parameter is defined as $k_{2}$ (ES→P) from Scheme 1. In agreement with this parameter, the mixtures given by CG (25%w/w) and CU (10 and 25%w/w) showed the best trends to departure of the product from the ES complex. Figure 2 shows the relationships between $K_{M}$ vs. % w/w in each solvent mixture studied. $K_{M}$ is associated with the bound strength of the ES complex (affinity constant denoted by $K_{M}$). Thus, CG at 25%w/w shows a peak in comparison to the other mixtures, suggesting that the best trend to departure of the P from the ES complex will be those mixtures given by CU. Figure 3 shows the relationships between $\left(\frac{k_{\mathrm{cat}}}{K_{M}}\right)$ vs. %w/w in each solvent mixture studied. The best catalytic efficiency $\left(\frac{k_{\mathrm{cat}}}{K_{M}}\right)$ is located in CU at 25%w/w. Note that this composition is more effective than pure buffer phosphate evaluated through its $\left(\frac{k_{\mathrm{cat}}}{K_{M}}\right)$ and $K_{M}$ parameters.

These kinetic responses are focused on the physico-chemical properties of the mixtures, namely, polarity, ability to establish HB, density, and viscosity, among others. Note that, in DES, the main solvent–solvent interactions correspond to HB, and the nature of the solvent will be exerting an effect on the catalytic reaction as a result of its abilities to donate or accept HB. (See Table S4 in SM). Durand et al. reported the effect of water molecules over CU using the alcoholysis of phenolic esters with 1-octanol and CALB immobilized as a model [44,45]. On the other hand, previous studies showed that adding water to CU established strong HB as the main driving force. Those water molecules that interact with charged and polar residues of the enzyme promote its activity and stability [46]. Zhao et al. showed that a small amount of water, even only about 5%, increases the activity and selectivity of protease in glycol-DES [47].

The kinetic analysis of CALB in these studied reaction media showed two results a priori. First, high quantities of water from the phosphate buffer in the mixtures suggests high degrees of freedom and the solvation of each moiety of the DES. Note that the addition of buffer changes the structure of DES. Moreover, when there is the presence of water molecules above a certain %w/w, the phosphate buffer will be also fit into the HB networks of the DES components. Then, a strong HB network given by the reaction media could be immobilizing the enzyme through interactions between the water molecules and the charged and polar residues. This strong polar network could break the DES by a gradual loss of HB interactions between the components of the DES. Second, at low quantities of water in the mixtures, the water molecules are sufficient to establish interactions with the DES, and these molecules should be building some clusters embedded in the polar network [48]. Note that the catalytic efficiency at 25% w/w is improved by 10 times when it is changed into CG by CU and 6 times when is changed by CE. However, it improves the CE 60 times when it is changed by CU. Thus, the catalytic efficiency provides the following order: CU > CG > CE. Analyzing the different % w/w by CU demonstrates that 25% w/w is improved 7 times with respect to 10% w/w, but 25% w/w is stronger (20,000 times) with respect to 50% w/w. In summary, the best mixture over the catalytic reaction will be CU at 25% w/w. These insights provided by this study showed the importance of the water effect in DES-based reaction media. Furthermore, the results highlight the important potential for customizing water-in-DESs for optimal enzyme stability and activity. Note that the kinetic results shown in Figure 1 are in agreement with Nolasco et al. [49]. This report established that the addition of water induces structural changes in the DES, as evidenced by the changes in the reaction rates. This report specifies three main regimes, namely “low water”, “medium water”, and “high water” contents, where understanding the interactions between water and DES in the mixtures to evaluate the effects of DES on the solubility and stability of the reaction components is crucial [49]. Then, at 50% w/w, the interaction between the components of the DES is weak, and adding more water from the phosphate buffer led to the progressive reduction of the DES-DES interaction until they became similar to an aqueous solution. On the other hand, at 10% w/w, the kinetics performed could be correlated with an aqueous solution, which shows the lower kinetic parameters. On the other hand, Kim et al. explored the relationships between the activity and conformational changes of CALB and the properties of some conventional organic solvents and ionic liquids, indicating that the structure of the catalytic cavity is solvent dependent. The molecular dynamic (MD) integrated into experimental studies showed that not all solvents can induce the open conformation of the catalytic cavity, suggesting that the enzyme activity depends both on the open conformation of the catalytic cavity and its size [50].

In order to examine the solvent effects on the catalysis behavior, each mixture was analyzed in great detail to consider the physicochemical and solvent effects parameters. In general terms, the kinetic parameters (see Table S4 in SM) showed that, with low amount of DES in the mixtures, their kinetic parameters tend to increase with increasing conductivity. However, CU shows a drastic decrease in the catalytic affinity parameter with increasing DES composition, suggesting the key role of the composition of the environment over the catalytic reaction. Thus, aqueous-DES mixtures applied in biocatalysis take advantage due to tunable features, which will allow the development of suitable solvents for applications when reagents cannot be dissolved well [36,38,39,40,41].

The viscosities of all the mixtures studied are very similar in a broad range of % w/w. (See Table S5 and Figures S7–S9 in SM). This parameter, in general terms, begins slowly to change, since 85% w/w shows a peak at 100% w/w for CU. Note that, at room temperature, most of the DES are highly viscous in comparison to its aqueous mixtures [18,51]. This fact is attributable to the nature of the components, temperature, the presence of water in the mixture, and the HB network between the components [52]. A more detailed inspection of each Figure shows three regions perfectly delimited. For example, CU shows: (i) 10–75% w/w with viscosity values between 0.003–6.40 (mPa·s); (ii) 85–95% w/w with viscosity values between 18.38–158.30 (mPa·s); and (iii) 98–100% w/w with viscosity values between 360.00–1006.30 (mPa·s). CG shows three regions: (i) 10–65% w/w , with viscosity values between 0.22–7.78 (mPa·s); (ii) 75 to 90 % w/w, with viscosity values between 15.69–48.10 (mPa·s); and (iii) 90–100% w/w, with viscosity values between 48.10–361.29 (mPa·s). Finally, CE shows: (i) 10–50% w/w, with viscosity values between 0.63–3.66 (mPa·s); (ii) 55–85% w/w, with viscosity values between 3.66–30.60 (mPa·s); and (iii) 85–95% w/w, with viscosity values between 30.60–73.42 (mPa·s). These ranges of % w/w could be associated with the strength and nature of the interactions and how the water molecule is associated in the network of DES. The third region associated with a low amount of water is the region of predominating interactions of DES-DES and the water molecules surrounding the network of DES. Change is evident with an amount of water added, suggesting the entry of water molecules into the network (DES-H_2_O-DES). Finally, the water molecules integrate into the network and interact with the HBD and HBA moieties separately. In this region, the component of the DES should be fully solvated by water molecules, suggesting an aqueous solution of this DES. (See Scheme S2 in SM) [32,44] On the other hand, it must be highlighted that water molecules play a key role within the lipase activity, enhancing its flexibility and reactivity [44,53].

**Figure 4:**
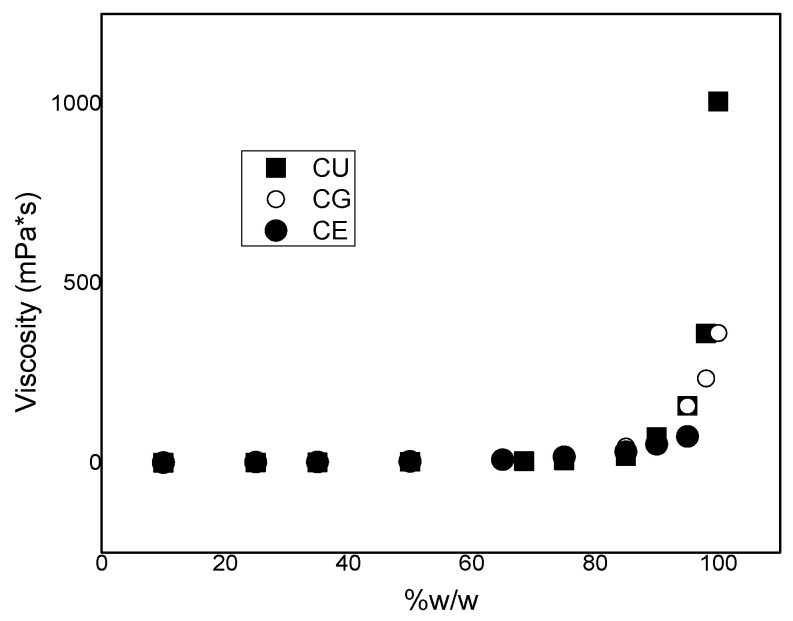
Relationship between viscosity of the studied DES and its mixtures vs. % w/w.

Figure 4 shows the relationships between the viscosity of the studied DES and its mixtures vs. % w/w. Dai et al. studied the water effect on the structure and physicochemical parameters of the DES, suggesting that, below 50% *v*/*v* water content, the DES structure is maintained [54]. Figure 4 shows a constant viscosity measurement in the whole range between 10–80% w/w, suggesting that water has an effect on DES-DES interactions, breaking the network that these molecules form and affecting the viscosity of the pure DES. The water molecules added into a DES form HB and influence the molecular arrangement, enhancing or disrupting molecular organization and impacting the properties of the solvent. Further dilutions establish solutions of the free forms of the DES components in water [51,54]. Shehata et al. showed an integrated study based on thermoalkalophilic lipases in reline with varying hydration levels, suggesting an enhanced mobility of the lid domain in softly hydrated reline [55]. Finally, the high viscosity of DES is attributed to its unique molecular structure and interactions, promoting limitations and advantages, e.g., offering stability for chemical reactions and supporting enzyme immobilization.

**Figure 5:**
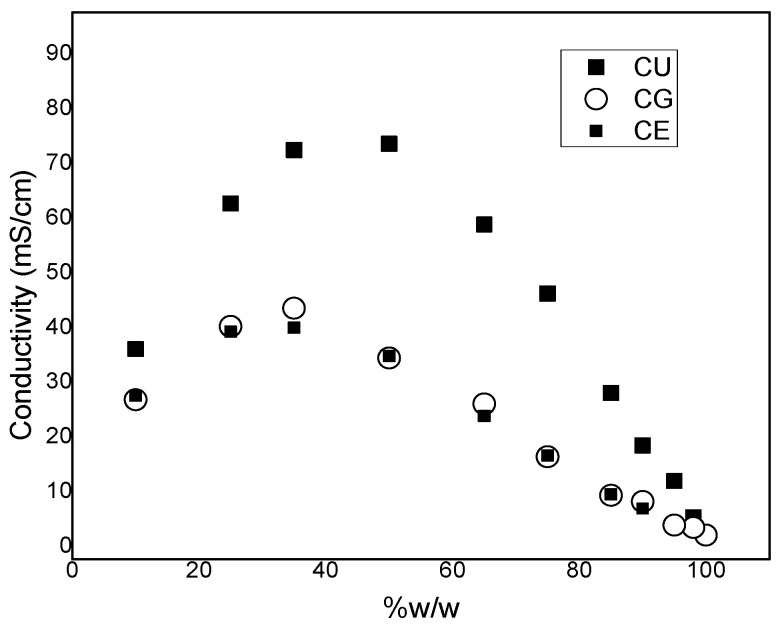
Relationships between conductivity of the studied DES and its mixtures vs. %*w*/*w*.

On the other hand, the conductivity parameter in each DES for the whole range of % w/w studied is demonstrated in Table S6 and Figures S10–S12 in SM. Figure 5 shows a similar behavior for CG and CE, but CU is higher than the others, showing a maximal value of conductivity close to 70 ($\frac{\mathrm{mS}}{\mathrm{cm}})$ located between 35 and 50%*w*/*w*. However, the maximal value for the other studied DES is close to 40 ($\frac{\mathrm{mS}}{\mathrm{cm}})$, between 25 and 35%*w*/*w*, suggesting that its maximal value could be associated with the nature of the DES. In general, the conductivity increases with the amount of water added, and then, it decreases as the % DES increases in the mixture. A high % DES shows low conductivities, suggesting strong DES-DES interaction strength that are associated with high values of viscosity [18,51,54,56].

The relationships between conductivity vs. viscosity in the whole range of % w/w are shown in SM for each studied DES (see Figures S28–S30 in SM). CU shows that, as the amount of DES in the mixture increases, the conductivity increases until reaching a maximum value, which then declines to increase the density of the mixture in the vicinity of the pure DES. CG shows that, as the amount of DES in the mixture increases, the conductivity decreases until it reaches a minimum value, and then, the density of the mixture increases in the vicinity of the pure DES. CE shows that, starting from maximum conductivity at a low proportion of DES in the mixture, this value is maintained and increases the density of the mixture in the vicinity of the pure DES. Previous works have reported that most DESs show low ionic conductivity. However, mixtures of DES/water have demonstrated that the addition of water increases the conductivity [18,51,54,56]. Note that, for each mixture where the kinetic study was performed, the conductivity parameter is higher in comparison to the viscosity parameter, suggesting greater movements and the development of charges and higher abilities to establish HB.

Another analyzed parameter was the acidity of each reaction media, which was measured using p-nitrophenol dye. (See Table S7 and Figures S13–S15 in SM). Acidity is defined as the ability of an aqueous solution to resist a change of pH [42,57]. CU shows two zones. One of them, rich in DES where the phenolate form at $\lambda_{\max}$ close to 400 nm, was found, while mixtures rich in water showed a protonated form (phenol) at $\lambda_{\max}$ close to 220 nm. Note that, between 50 and 75%*w*/*w* had a gap, suggesting a phenol–phenolate equilibrium. On the other hand, CG shows this gap at % w/w90, while CE does not show changes in the studied mixtures, suggesting its protonated form of the dye in the last DESs. These results suggest that CU is more sensitive toward solvation effects in comparison with the other DES. Figures S31–S33 in SM show the relationship between conductivity and the acidity in the studied DES and its mixtures. For instance, in CU, as the proportion of DES in the mixture increases, the conductivity increases. And, it is associated with the presence of the protonated probe, suggesting the ability to establish HB in mixtures enriched with water.

In order to interpret the solvation effects, mixtures were evaluated for their solvatochromic parameters in each studied reaction media using the well-known Kamlet–Taft model [58,59,60] based on a multivariate empirical equation that includes the following parameters: Nile Red, hydrogen-bond acidity (α), measuring the ability of the reaction media to donate an HB to the substrate hydrogen bond basicity (β), measuring the ability of the reaction media to accept a HB from the substrate. See the details in SM Tables S8–S10.

Nile Red (NR) is a neutral solvatochromic dye that exhibits a large positive solvatochromism, going from non-polar to polar reaction media (see Figures S16–S18 in SM) [61]. Then, Nile Red displays increasing red shifts in both absorption and emission wavelength as the solvent polarity is increased. CU and CE show an evident change between 10 to 45% w/w for CU and 10 to 25% w/w for CE denoting a polar environment. However, CG does not show a change. These results indicate that CU and CE establish a different environment in comparison to CG. This environment suggests that mixtures of DES-water (with a high water content) cause a dramatic change in the structure of DES resulting from the rupture of the HB network formed between both moieties of the DES. This polarity is absolutely dependent on the DES type.

Figures S19–S21 in SM show the relationship between α vs. % w/w. CU shows two strongly defined zones: (i) 10 to 50% w/w, where the ability of the reaction media to donate a HB to the probe increases, and (ii) 65 to 100% w/w, where the ability of the reaction media to donate a HB to the probe diminishes. CU at the first range shows a high value of the α parameter, suggesting that the region associated with high quantities of water molecules establishes a strong network by HB interactions, increasing the possibility of donating HD, and the DES must be absolutely solvated, proposing a solution of DES in this range of % w/w. Then, the enzymatic performance is increased, when the reaction media is able to donate HB toward the E and/or ES complex (highly polar range). CG and CU show similar behavior to CU.

On the other hand, the β parameter is shown in SM through Figures S22–S24. CU shows two trends: 10–85% w/w, where the ability of the reaction media to accept an HB from the substrate decreases, and then it increases above 85–100% w/w. On the other hand, CG and CE show two trends with different % w/w. For instance, CE shows (i) 10–35% w/w, and (ii) 50–100 % w/w decreases the ability of the reaction media to accept an HB from the substrate. In relationship to CU at 25% w/w, β shows a low value, suggesting that the E and/or ES complex are not able to donate HB toward the environment.

Finally, the polarity determination was performed by monitoring the spectroscopy behavior of Reichardt’s dye (see Figures S25–S27 in SM). The solvatochromic parameter $ET_{30}$ is based on the intermolecular charge-transfer absorption band of 2,6-diphenyl-4-(2,4,6-triphenyl-N-pyridino) phenolate [62]. The relationships between $ET_{30}$ and % w/w for CU show high polarity at the beginning, suggesting interactions such as water–water; reacting–water, and DES-water; this parameter diminishes significantly (from 65% w/w), suggesting at the end DES-DES interactions. CG shows a constant behavior in the whole range of mixtures. Finally, CE showed two delimited region, located at %w/w >35, highly associated with the high polarities given by HB established by the moieties of DES in comparison to the range, and between %*w*/*w* > 10 and %*w*/*w* < 35, wherthe increased value of $ET_{30}$ is influenced by the increase of DES in the mixture.

In summary, the aqueous CU mixture at 25% w/w, where the lipase activity reaches its best behavior, is clearly a mixture with possibilities for establishing HB given by the reaction media (high amount of water molecules), conferring a polar environment and with high conductivity. The density of this mixture was determined by using an Anton Paar Density Meter Instrument DMA 35 (δ = 1.047 g/mL) with this aqueous-CU mixture at 1.008 M. At this concentration, the DES structure is disrupted; water–water and DES-water interactions dominate [43]. Then, at those dilutions, the interactions between HBA and HBD should be broken, suggesting aqueous solutions of the single DES components. The stabilized network from HB given by water molecules surrounds the DES, and those bonds formed between the reacting of the DES will improve the enzymatic activity attributed to the conformational stability given by the reaction media [36]. Such conformational changes should be local changes on the enzyme and able to improve its activity. On the other hand, a low amount of water in DES suggests that the presence of water molecules weakens the CALB-DES interaction based on HB in the solvation shell, suggesting that CALB acquires a more restrictive structure in the presence of DES. Shao et al., in their study based on molecular dynamics, suggest that water molecules play a key role in the active site in mixtures DES/water [36]. In addition, aqueous DES mixtures effectively influence the activity of CALB, highlighting the % w/w which improves its kinetic performance. Notably, the binding site of CALB responsible for the catalytic activity, is composed of serine (Ser105), histidine (His224), and aspartate (Asp187) residues [50,63,64]. This catalytic triad is located at the bottom of a deep and narrow cavity which is accessible for the solvent [50]. The aperture to the catalytic cavity, where substrates bind to the catalytic triad, is the path that permits substrate entrance and product departure. In light of our results, the high activities achieved at 25% w/w are presumably due to HB formed with the amino acid residues of CALB. Then, the lower activities observed at higher %*w*/*w* of DES must be associated with either a harmful effect that the DES components may have on the enzyme or because of the loss of non-conventional nature of the DES/water blend [65,66]. These results show that the environment in the cavity changes for different solvent compositions, suggesting that the kinetic response is dependent of the bulk properties of the solvent. This result highlights the relevance of a protic reaction medium on the catalysis. Note that, solvent effect studies have been addressed experimentally in order to get detailed information about the phenomena that are not observable and occurring at microscopic level. In summary, the kinetic data suggests that the catalytic activity could be established by a hydrogen atom from the reaction media or between an acidic hydrogen atom from the catalytic cavity and the solvent. Finally, a more detailed analysis of the solvatochromic parameters showed that, in the region where it is performed, the kinetic study opens the possibility of establishing HB from the media toward the substrate, with (α) being CU and its mixtures of the solvent with the greatest potential to establish HB with the other reaction media. On the other hand, β parameter is observed in CU and CG and their mixtures, but only in the first region for CG (10–45% w/w). The polarity denoted by the Nile Red and ET_30_ parameters [67] shows that the region with enhanced polarity is that given by CU between 10–45% w/w. Then, the region surrounded by 50% w/w could be denoted like a region where the properties of the reaction media are changing.

## 3. Materials and Methods

### 3.1. Materials

Urea (purity ≥ 99% (HClO_4_ titration)), choline chloride (purity ≥ 99% (thin layer chromatography, TLC) and water content ≤ 5%), 1,2,3-propanetriol (purity ≥ 99.5% (gas chromatography, GC)), 1,2-propanediol (purity ≥ 99.5% (GC)), p-NPL (purity ≥ 98% (GC)), p-nitrophenol (p-NP, purity ≥ 99% (GC)), dimethyl sulfoxide (DMSO, purity ≥ 99.9% (GC)), N,N-diethyl-4-nitroaniline (purity ≥ 99% (GC)), p-nitroaniline (purity ≥ 99% (GC)), Reichardt’s dye content 90%, and Nile Red were purchased from Sigma, Saint Louis, MO, USA. The salts, KH_2_PO_4_, and K_2_HPO_4,_ were acquired from Merck (purities (alkalimetric assay) >99.5%). All reagents were used as soon as delivered. For the preparation of the aqueous solutions used, ultrapure water was used (Merck-Millipore Simplicity™ UV water purification system, Millipore SAS, Molsheim, France). CALB was purchased from Sigma Aldrich as lipase from *Candida antarctica* (lyophilized powder). The water standard was 1%, with certified reference material for Karl Fisher titration, titrant 5 for volumetric Karl Fisher titration with two component reagents (contains methanol, iodine), and solvent for volumetric Karl Fisher titration with two component reagents (contains methanol, imidazole).

### 3.2. Preparation of Deep Eutectic Solvents

The preparation of DES based on choline-urea, choline-1,2-propanediol, and choline-1,2,3-propanetriol were achieved by combining HBA (choline chloride) and the HBD. Choline chloride is the most used HBA, which is combined with suitable HBD species by heating the mixture in different molar ratios at temperatures between 80 °C and 110 °C under constant stirring until a clear liquid mixture is observed [68,69]. After dissolving both the HBD and the HBA, the resulting solutions were clear stable liquids. Irrespective of the preparation, the DESs were monitored for at least a week, after which only the liquids with no apparent change in their physical characteristics (opacity, precipitation, etc.) were used in further studies. The studied DESs were prepared in the following proportions: choline-urea (1:2), choline-1,2,3-propanetriol (1:2), and choline-1,2-propanediol (1:2). These molar ratios were selected in order to maintain the stability of the mentioned DESs and their mixtures.

### 3.3. Mixtures

This study used pure phosphate buffer as the aqueous media 50 mM and pH = 7.0 and DES/buffer mixtures in a wide range of % w/w with respect to each studied DES (see data in Supplementary materials). Each mixture was prepared by weighing the proper amount of DES and buffer into a screw-capped vial. To favor mixing, each mixture was shaken for 1 min, then left to equilibrate overnight before use in order to equilibrate the mixture. All of the studied mixtures appeared homogeneous after this treatment [14]. The rheological properties of the DES were modified by changing the % w/w by mixing the DES with limited amounts of buffer, resulting in solvents with varying viscosities, ranging from clear-fluid liquids to a clear viscous phase. Note that all these mixtures were used in the kinetic study and the solvent effects analysis.

### 3.4. Lipase Activity Assays

This study used each DES and buffer phosphate as aqueous media and DES/buffer mixtures in a wide range of % w/w. The substrate solution (p-NPL) was prepared in DMSO at 58 mM and it (10 μL) was directly injected in each reaction media. The enzymatic solution was prepared by adding 10 mg to 1 mL of potassium phosphate buffer solution. Lipase activities were carried out by UV-vis spectrophotometry using an Agilent 8453 UV-Vis spectrometer connected to a recirculatory water bath at 25 °C. Aliquots from a stock solution (50 μL) of lipase were added to 1 mL each reaction media containing the p-NPL. The release of p-NP was recorded by following the increase in absorbance at 410 nm. Initial reaction rates were calculated during the first 150 s of the initial segment of the reaction profiles [14,17].

The determination of the Michaelis–Menten kinetic parameters ($k_{\mathrm{cat}}\;$and $K_{M}$) was performed according to Equation (1):

(1) $$v=\frac{k_{\mathrm{cat}}\left[E_{0}\right]\left[S\right]}{K_{M}+\left[S\right]\;}$$

where v corresponds to the rates of CALB-catalyzed hydrolysis of p-NPL. $\left[E_{0}\right]$ is the enzyme concentration used in the hydrolysis experiments, and $\left[S\right]$ corresponds to the substrate concentration at which the associated reaction rate was determined [14,17].

### 3.5. Electric Conductivity Measurements

The conductivity of the samples was measured at room temperature using an AD3000 EC/TDS Temperature Meter provided with a 4-pole conductivity probe, expressed in $\frac{\mathrm{mS}}{{\mathrm{cm}}^{-1}}$ within an uncertainty of ±0.01 $\frac{\mathrm{mS}}{{\mathrm{cm}}^{-1}}$. All measurements were performed in duplicate [14,18,51].

### 3.6. Viscosity Measurements

Viscosity parameter (δ) expressed in (mPa·s) was used to characterize the reaction media and to evaluate their effects on the enzymatic reactions. The dynamic viscosities were measured at 25 °C using a rotational viscometer (Fungilab Viscolead series) connected to a recirculatory water bath. Each measurement was performed in duplicate for each studied reaction media [18,51].

### 3.7. Acidity Measurements

Into a quartz cuvette of an optical path of 1 cm, 975 μL of each mixture were injected with the dye p-nitrophenol (p-NP) previously prepared in methanol. Twenty-five μL of the stock solution of p-NP were evaporated to dryness for 30 min in a rotary evaporator Buchi R-300 (Büchi, Flawil, Switzerland). The concentration of stock solution was 1.85 × 10^−3^ M. Each reaction media was thermostated at 25 °C and measured using a UV-vis spectrophotometry Agilent 8453 UV-Vis spectrometer (Agilent Technologies, Inc. Santa Clara, CA, USA) [57].

### 3.8. Solvatochromic Solvent Parameters

The Kamlet–Taft correlation quantifies solute–solvent interactions that are defined as specific (HB or donor-acceptor interactions) and non-specific (polarity/polarizability) effects. This scale considers 4 chromophores to evaluate the solvent properties, namely Reichardt dye (RD), N,N-diethyl-4-nitroaniline (B), p-nitroaniline (C), and Nile Red (NR) [58,59,60]. These solvatochromic probes undergo spectral shifts, making it possible to determine the nature of the solvent in the solvation shell using Equations (2)–(6) below.

These parameters were measured by injecting the reaction media (950 μL) into a quartz cuvette with an optical path of 1 cm with the probes, previously prepared in methanol. Note that 50 μL of the stock solution of each probe was evaporated to dryness for 30 min in a rotary evaporator Buchi R-300. The concentration of stock solution of each probe was 1.85 × 10^−3^ M. Each reaction media was thermostated at 25 °C and measured using a UV-vis spectrophotometry Agilent 8453 UV-Vis spectrometer [14,70].

(2) $${\mathrm{ET}}_{30}=\frac{28591}{\lambda_{\max}\;\left(\mathrm{RD}\right)}$$

(3) $$\;\pi^{*}=0.314\;\left(27.52-v_{B}\right);\;$$

(4) $$\alpha =0.0649{\;\mathrm{ET}}_{30}-2.03-0.72\pi^{*}$$

(5) $$\beta =\frac{\left(1.035{\;\nu }_{B}+2.64-\nu_{C\;}\right)}{2.80}$$

(6) $$E_{\mathrm{NR}}=\frac{1.196\times 10^{5}}{\lambda_{\max}\left(\mathrm{NR}\right)}$$

### 3.9. Karl Fischer Method

The measurements were performed in a Hanna HI 933-02 Karl Fischer Volumetric Titration apparatus. This titration was used to obtain reference water concentration. Karl Fisher titrations were carried out by filling the beaker titration up with solvent (50 mL). The syringe is filled and needled with the sample. Then, it is weighed, and the sample is added to the beaker titration. The syringe is weighed again in order to determine the added sample mass (by the difference of the two measurements). The exact weight is entered in order to start the analysis. Then, for this study, the water concentration determined for CU at 98% *w*/*w* was 2.205% *w*/*w* in water (2.170 and 2.240% *w*/*w* in water); CG at 100% w/w was 0.783% w/w in water (0.765 and 0.800% w/w in water), and CE at 90% w/w was 11.700% w/w in water (11.600 and 11.800% w/w in water), respectively [71]. Note that the studied DESs will contain a certain amount of water which can be considered as an impurity, especially in hydrophilic DES, even if they are dried.

## 4. Conclusions

This paper presents a kinetic analysis of the enzymatic activity of CALB in three DESs. This reaction has been selected as a model system for analyzing the solvent effect given by different % w/w of mixtures of DES/buffer and its effect on the enzymatic reaction. Note that this type of reaction cannot be performed in pure DES. However, mixtures of DES/buffer had better kinetic performance than pure phosphate buffer. Thus, mixtures of DES/buffer are a good model to study the solvent effect on an enzymatic reaction. The results show that changes in the composition of the reaction media determine the activity of CALB, with the CU at 25% w/w the best mixture in comparison to the others. This result is explained in terms of the kinetic study and the physico-chemical and solvent effects parameters where the nature of the reaction media sometimes did not allow the reaction performance. However, in this case, the enzymatic reaction requires a reaction medium rich in water molecules to allow for HB formation, suggesting an interaction between the substrate and the enzyme’s active site, which is able to promote the high degrees of freedom that determine the best enzyme conformation in comparison to mixtures rich in DES. This research emphasizes the importance of solvent effect analysis and opens a complete discussion of biocatalysis, complemented with kinetic data and suggesting the features of the environment that improved the catalytic performance.

## Data Availability

All data are reported in the SMs.

## Supplementary Materials

The following supporting information can be downloaded at https://www.mdpi.com/article/10.3390/molecules29143296/s1. Ref. [72] is cited in Scheme S2.
