# Supplementary material for: Analysis of the Behavior of Deep Eutectic Solvents upon Addition of Water: Its Effects over a Catalytic Reaction
Source: Molecules. 2024 Jul 12;29(14):3296. doi: 10.3390/molecules29143296 (PMC11279026; doi:10.3390/molecules29143296)
Supplement: Supplementary file 1 [file molecules-29-03296-s001.zip › molecules-3033086-supplementary.pdf]

molecules

MDPI

Supplementary Materials

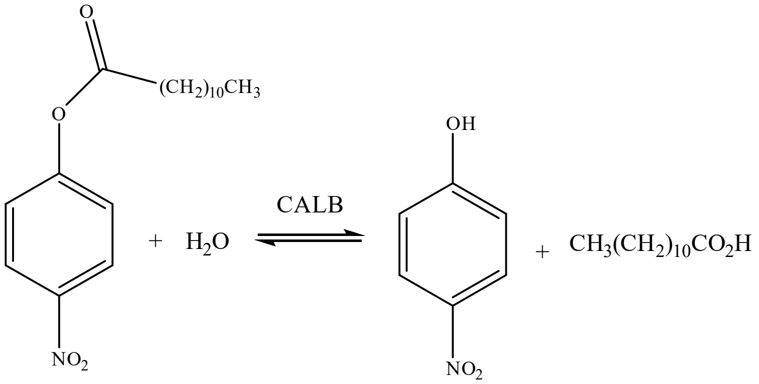

Scheme S1. General picture of the hydrolysis reaction of the substrate *p*-nitrophenyl laureate (*p*-NPL) mediated by CALB.

Table S1. Kinetic parameters determined in choline-urea (CU) and its mixtures (% w/w) for the CALB – *p*-NPL system.

1/vₒ	1/[S]	1/vₒ	1/[S]	1/vₒ	1/[S]
(s^{-1})	(M^{-1})	(s^{-1})	(M^{-1})	(s^{-1})	(M^{-1})
CU 10% w/w		CU 25% w/w		CU 50% w/w	
53300	1080.0	38200	2758.9	9240	2758.9
33000	956.0	23100	2069.2	6360	2069.2
22800	827.7	18200	1655.4	5680	1655.2
25500	864.0	14300	1379.5	5850	1675.9
14500	706.9	11500	1182.4	3870	1134.0
83500	1402.0	11600	1034.6	3120	827.7
		7550	827.7	3320	864.0

Table S2. Kinetic parameters determined in choline-1,2,3-propanetriol (CG) and its mixtures (% w/w) for the CALB – *p*-NPL system.

1/vₒ	1/[S]	1/vₒ	1/[S]
(s^{-1})	(M^{-1})	(s^{-1})	(M^{-1})
CG 10% w/w		CG 25% w/w	

118000	1661.5	69800	1944.1
84500	1301.3	54500	1555.2
72800	1115.4	48000	1296.0
58800	976.0	39500	1110.9
52200	780.8	32800	972.0
44000	709.8	30700	777.6
		25600	706.9

Table S3. Kinetic parameters determined in choline-1,2-propanediol (CE) and its mixtures (% w/w) for the CALB – *p*-NPL system.

$1/v_o$	1/[S]
(s^{-1})	(M^{-1})
CE 25% w/w	
24622.05	2592.07
22214.82	1944.05
15419.49	1296.04
14315.78	1110.89
10677.37	777.62
12605.41	864.02
11144.54	706.93

Table S4. Michaelis – Menten parameters determined for the CALB – *p*-NPL system in CU and its mixtures.

%w/w		$1/v_{max}$	K_M/v_{max}	v_{max}	K_M	k_{cat}	k_{cat}/K_M
		$(M/s)^{-1}$	(s)	(M/s)	(M)	(s^{-1})	(M^{-1} s^{-1})
10	CU	62110.98	103.70	$1.61E^{-5}$	$1.70E^{-3}$	$5.40E^{-3}$	3.18
25	CU	5925.13	15.21	$1.7E^{-4}$	$2.60E^{-3}$	$5.60E^{-2}$	21.54
50	CU	543.09	3.06	$1.80E^{-3}$	$5.50E^{-3}$	$5.50E^{-6}$	$1.10E^{-3}$
10	CG	14803.94	79.85	$6.7E^{-5}$	$5.30E^{-3}$	$5.40E^{-3}$	1.02
25	CG	1366.11	34.92	$7.3E^{-4}$	$2.50E^{-2}$	$5.30E^{-2}$	2.24
25	CE	5602.64	7.72	$1.8E^{-4}$	$1.40E^{-3}$	$5.00E^{-7}$	$3.60E^{-4}$

Table S5. Viscosity parameters determined in each studied DES and its mixtures.

%w/w	Viscosity	%w/w	Viscosity	%w/w	Viscosity
	(mPa*s)		(mPa*s)		(mPa*s)
CU		CG		CE	
90	71.70	90	48.10	90	51.66
75	6.38	75	15.69	75	16.66
50	2.49	50	2.32	50	3.66

25	0.59	25	0.65	25	1.76
10	3.00E^{-3}	10	0.22	10	0.63
35	0.76	35	1.79	35	2.29
68.5	4.26	65	7.78	65	8.51
85	18.38	85	44.98	85	30.60
95	158.30	95	158.03		
98	360.00	98	235.13		

Table S6. Conductivity parameter determined in each studied DES and its mixtures.

%w/w	Conductivity	%w/w	Conductivity	%w/w	Conductivity
	(mS/cm)		(mS/cm)		(mS/cm)
CU		CG		CE	
98	5.17	100	1.91	90	6.76
95	11.82	98	3.31	85	9.37
90	18.30	95	3.74	75	16.48
85	27.90	90	8.06	65	23.70
75	46.07	85	9.19	50	34.63
65	58.70	75	16.25	35	39.83
50	73.47	65	25.90	25	39.13
35	72.33	50	34.30	10	27.40
25	62.53	35	43.40		
10	35.93	25	40.07		
		10	26.67		

Table S7. λ_{max} determined in each studied DES and its mixtures for *p*-nitrophenol dye.

%w/w	λ_{max} (nm)	λ_{max} (nm)	λ_{max} (nm)
	CU	CG	CE
100	415	322	
98	410	315	
95	410	270	
90	400	230	228
85	390	230	228
75	390	230	228
65	395	230	228
50	230	230	228
35	225	230	228

25	225	225	228
10	210	225	228

Table S8. Solvatochromic solvent parameters determined in CU and its mixtures.

%w/w CU	NR	ET30	β	α	π*
100	893.64	102.11	7.19	7.80	-4.44
98	881.61	102.11	7.07	7.80	-4.44
95	881.61	102.11	7.07	7.80	-4.44
90	881.61	102.11	7.07	7.80	-4.44
85	885.59	102.11	5.99	7.80	-4.44
75	893.64	102.11	5.99	7.80	-4.44
65	893.64	102.11	5.99	7.80	-4.44
50	347.35	114.36	5.99	8.59	-4.44
35	343.11	114.36	5.99	8.59	-4.44
25	347.35	114.36	5.84	8.59	-4.44
10	407.04	114.36	5.84	8.59	-4.44

Table S9. Solvatochromic solvent parameters determined in CE and its mixtures.

%w/w CE	NR	ET30	β	α	π*
90	741.89	98.59	-0.56	3.47	1.25
85	741.89	98.59	-0.56	3.47	1.25
75	741.89	98.59	-0.56	3.47	1.25
65	741.89	98.59	-0.56	3.47	1.25
50	736.33	98.59	-0.56	3.47	1.25
35	733.58	98.59	-0.91	7.57	-4.44
25	333.22	96.92	-0.91	7.46	-4.44
10	333.22	95.30	-0.91	7.35	-4.44

Table S10. Solvatochromic solvent parameters determined in CG and its mixtures.

%w/w CG	NR	ET30	β	α	π*

100	959.02	95.30	-0.04	3.32	1.17
98	959.02	95.30	-0.18	3.32	1.17
95	959.02	95.30	0.09	3.32	1.17
90	959.02	95.30	0.22	3.32	1.17
85	959.02	95.30	0.35	3.32	1.17
75	959.02	95.30	0.35	3.32	1.17
65	959.02	95.30	0.35	3.32	1.17
50	959.02	95.30	6.95	7.35	-4.44
35	959.02	95.30	6.95	7.35	-4.44
25	959.02	95.30	6.95	7.35	-4.44
10	959.02	95.30	6.95	7.35	-4.44

Figure S1. Relationship between 1/vo vs. 1/[S] in order to determine the Michaelis-Menten parameters for the CALB – *p*-NPL system in 10 % w/w CU.

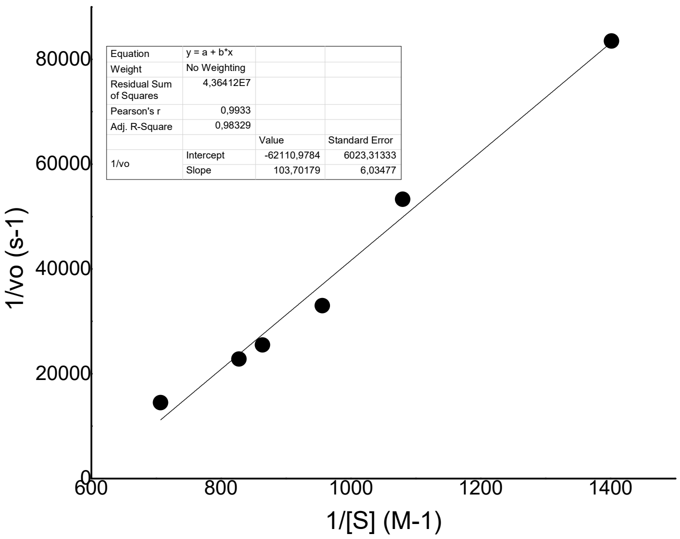

Figure S2. Relationship between 1/vo vs. 1/[S] in order to determine the Michaelis-Menten parameters for the CALB – *p*-NPL system in 25 % w/w CU.

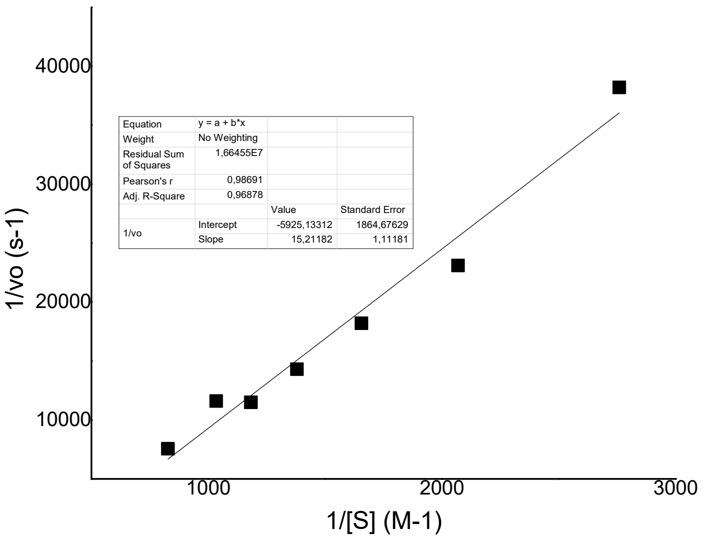

Figure S3. Relationship between 1/vo vs. 1/[S] in order to determine the Michaelis-Menten parameters for the CALB – *p*-NPL system in 50 % w/w CU.

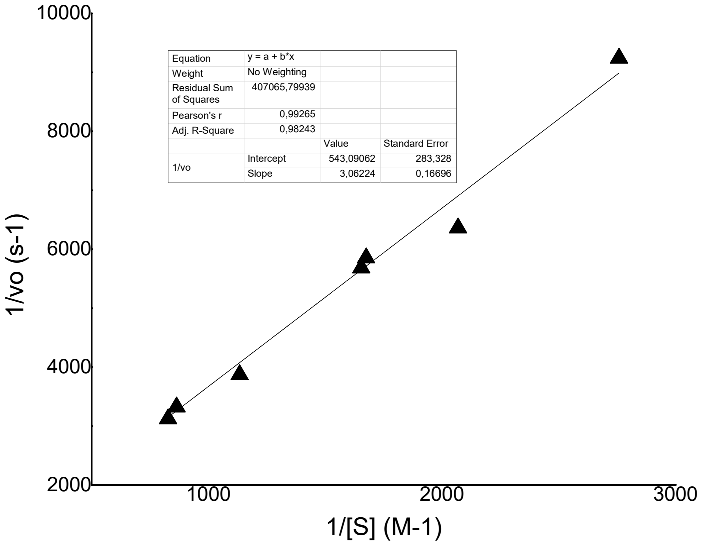

Figure S4. Relationship between 1/vo vs. 1/[S] in order to determine the Michaelis-Menten parameters for the CALB – *p*-NPL system in 10 % w/w CG.

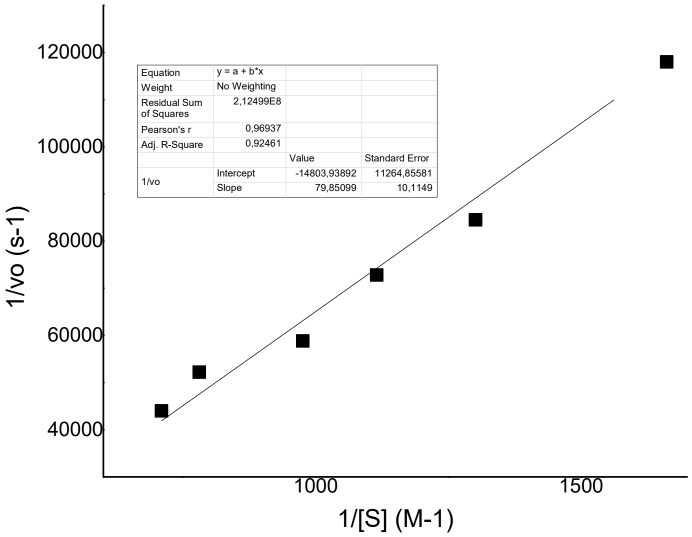

Figure S5. Relationship between 1/vo vs. 1/[S] in order to determine the Michaelis-Menten parameters for the CALB – *p*-NPL system in 25 % w/w CG.

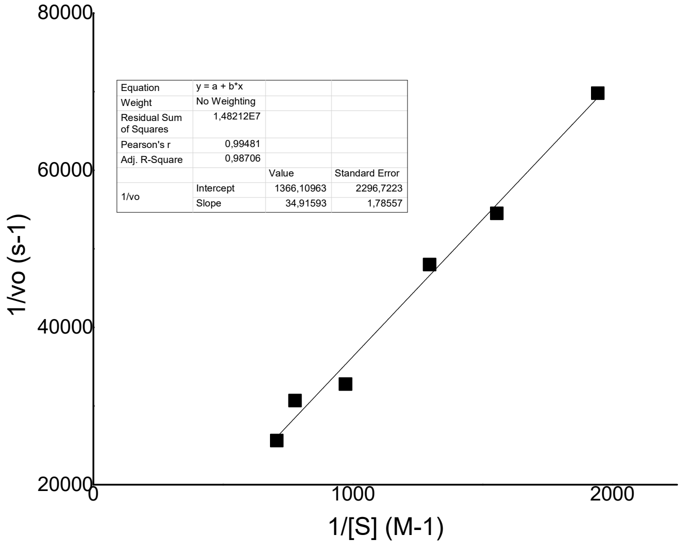

Figure S6. Relationship between 1/vo vs. 1/[S] inn order to determine the Michaelis-Menten parameters for the CALB – *p*-NPL system in 10 % w/w CE.

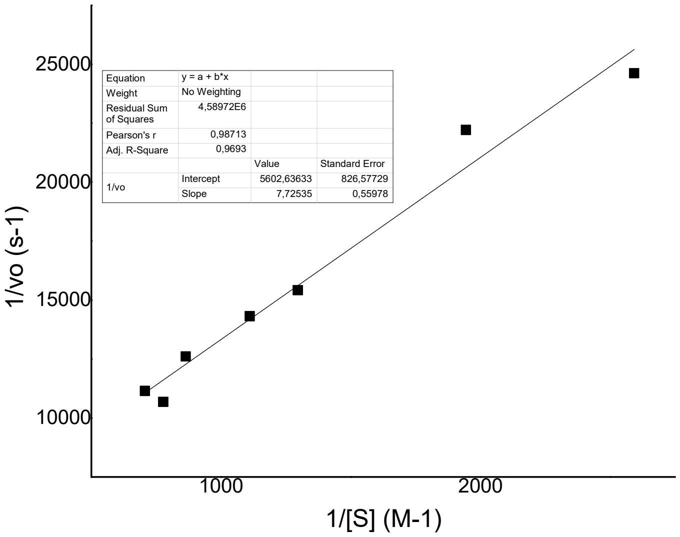

Figure S7. Relationship between viscosity vs. % w/w for CU.

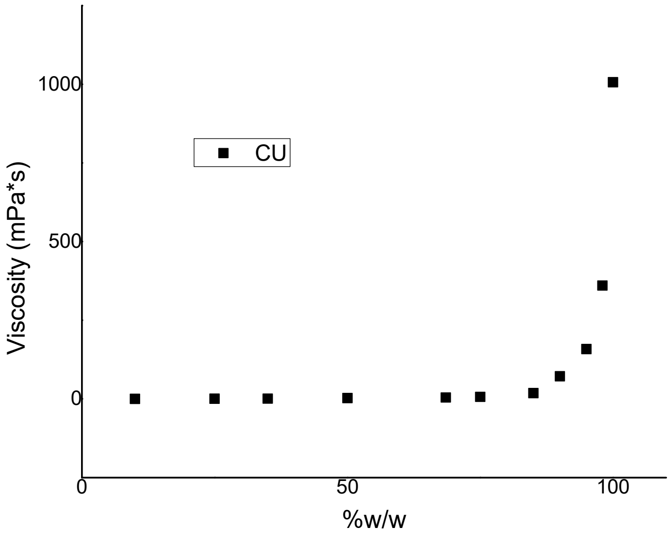

Figure S8. Relationship between viscosity vs. % w/w for CG.

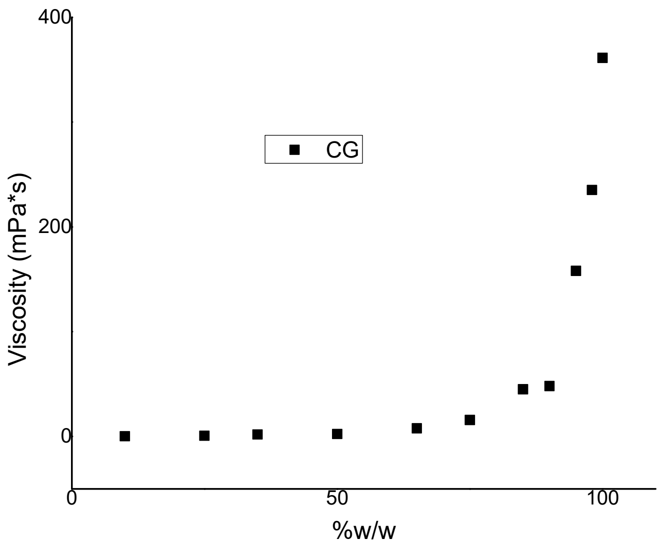

Figure S9. Relationship between viscosity vs. % w/w for CE.

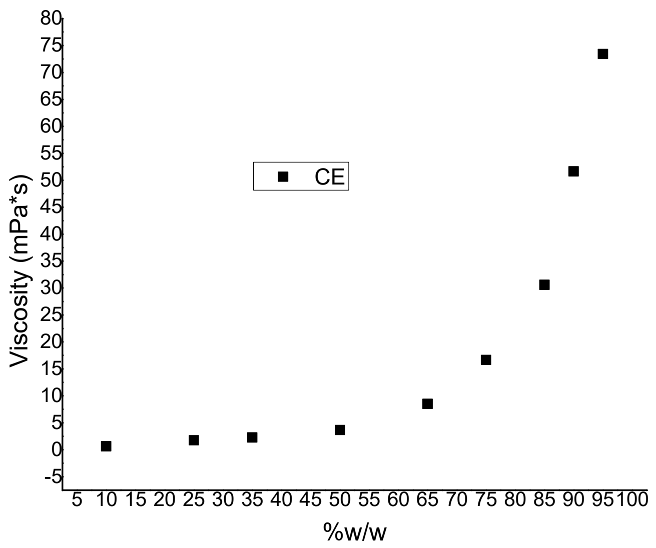

Figure S10. Relationship between conductivity vs. % w/w for CU.

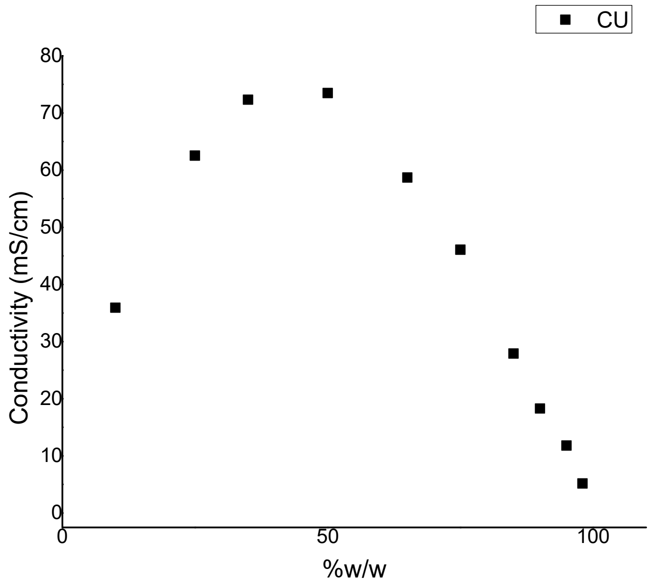

Figure S11. Relationship between conductivity vs. % w/w for CG.

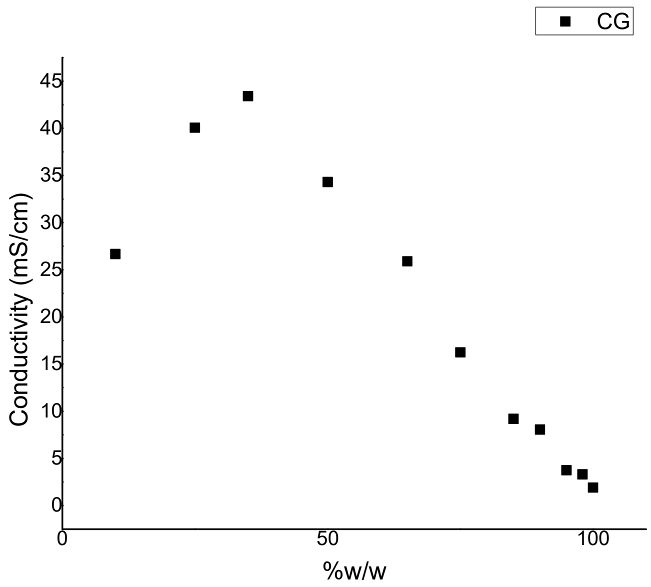

Figure S12. Relationship between conductivity vs. % w/w for CE.

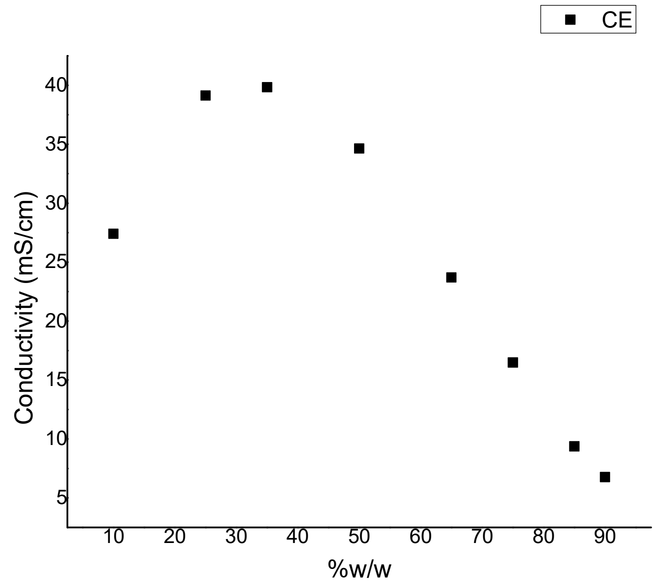

Figure S13. Relationship between λmax vs. % w/w for CU and its mixtures for *p*-nitrophenol dye.

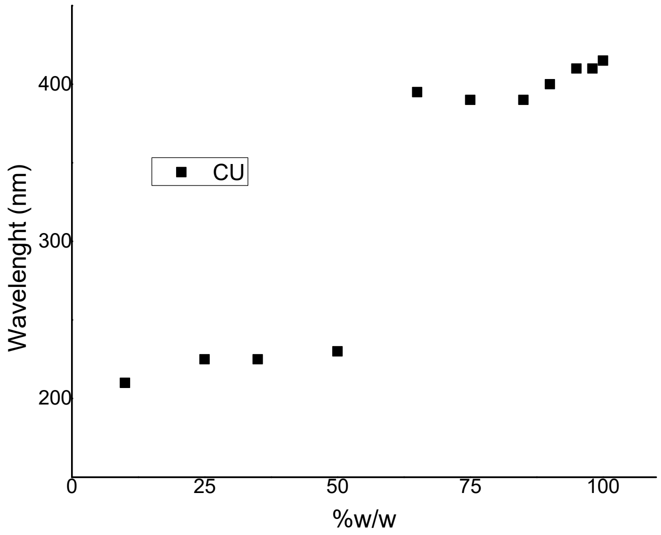

Figure S14. Relationship between λmax vs. % w/w for CG and its mixtures for *p*-nitrophenol dye.

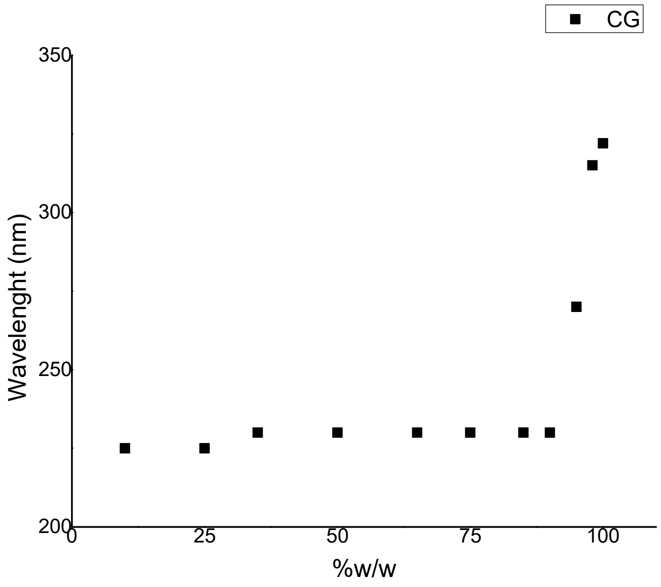

Figure S15. Relationship between λmax vs. % w/w for CE and its mixtures for *p*-nitrophenol dye.

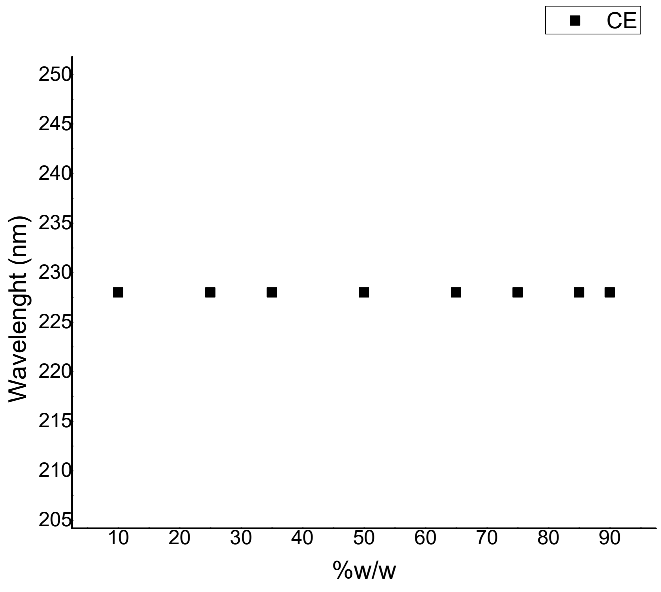

Figure S16. Relationship between NR vs. % w/w for CU and its mixtures for *p*-nitrophenol dye.

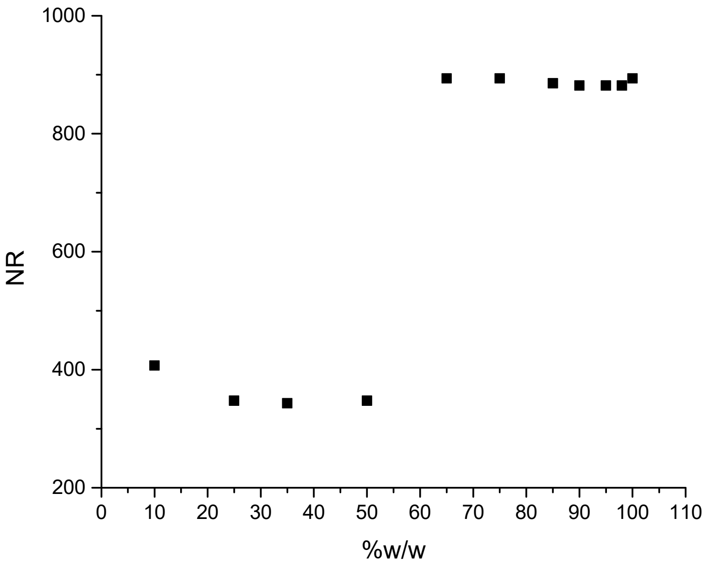

Figure S17. Relationship between NR vs. % w/w for CG and its mixtures for *p*-nitrophenol dye.

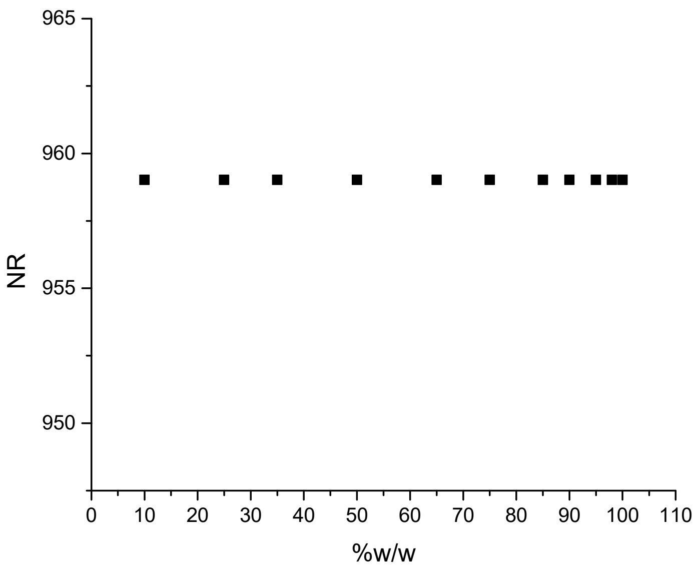

Figure S18. Relationship between NR vs. % w/w for CE and its mixtures for *p*-nitrophenol dye.

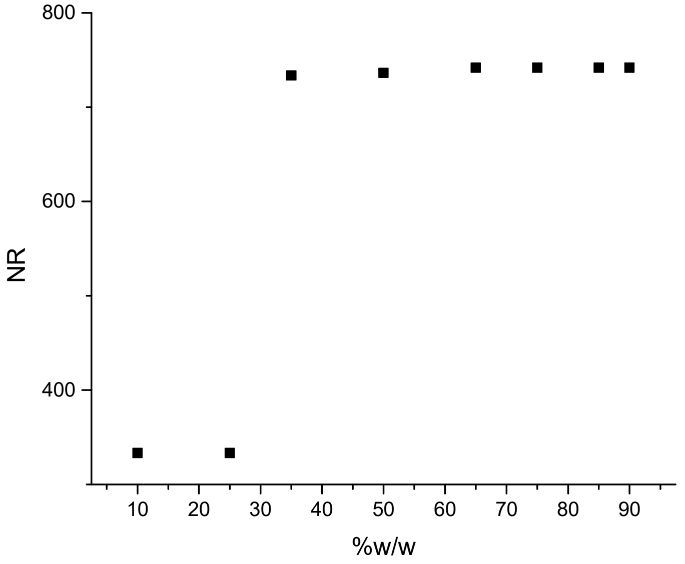

Figure S19. Relationship between α vs. % w/w for CU and its mixtures.

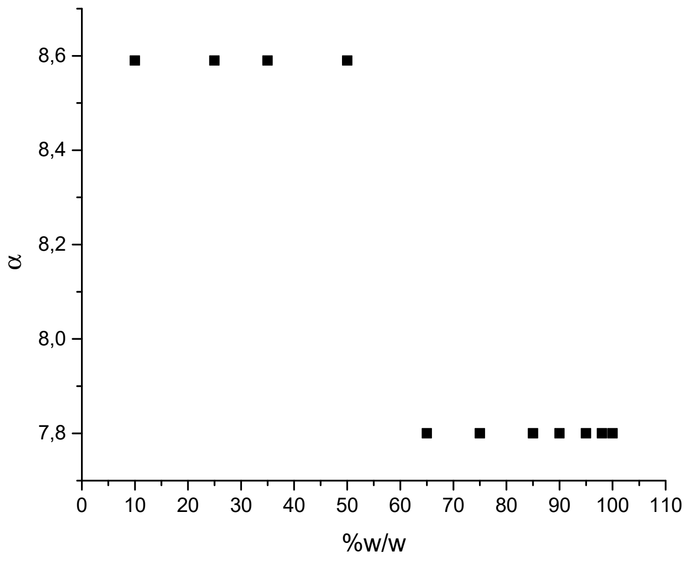

Figure S20. Relationship between α vs. % w/w for CG and its mixtures.

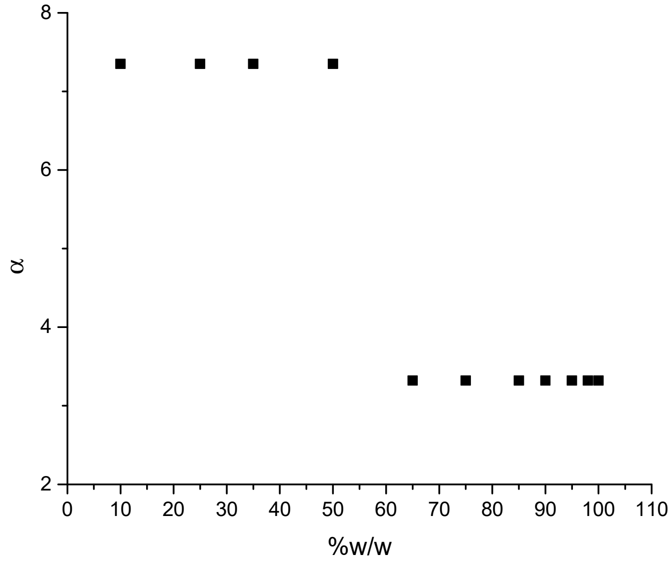

Figure S21. Relationship between α vs. % w/w for CE and its mixtures.

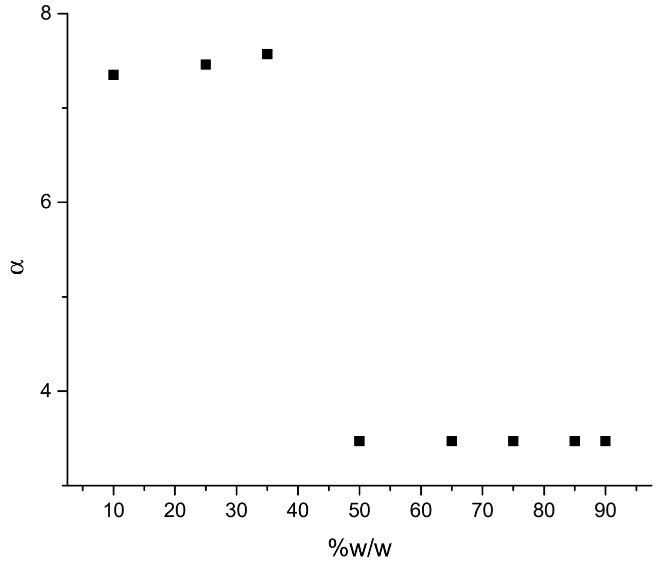

Figure S22. Relationship between β vs. % w/w for CU and its mixtures.

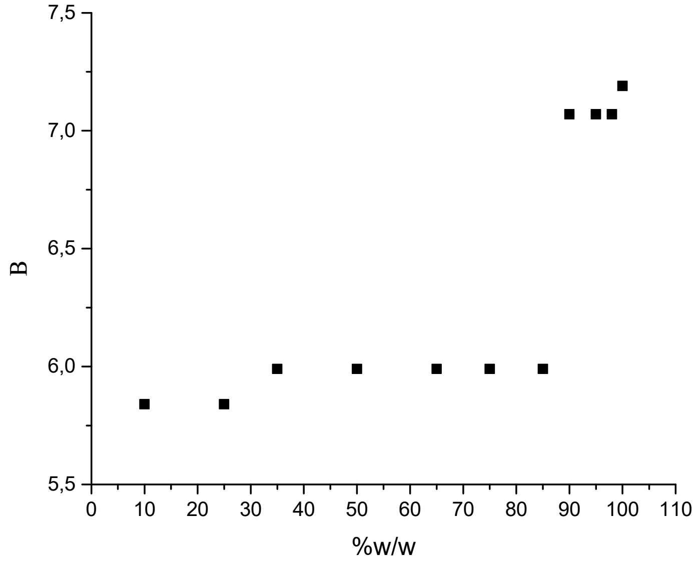

Figure S23. Relationship between β vs. % w/w for CG and its mixtures.

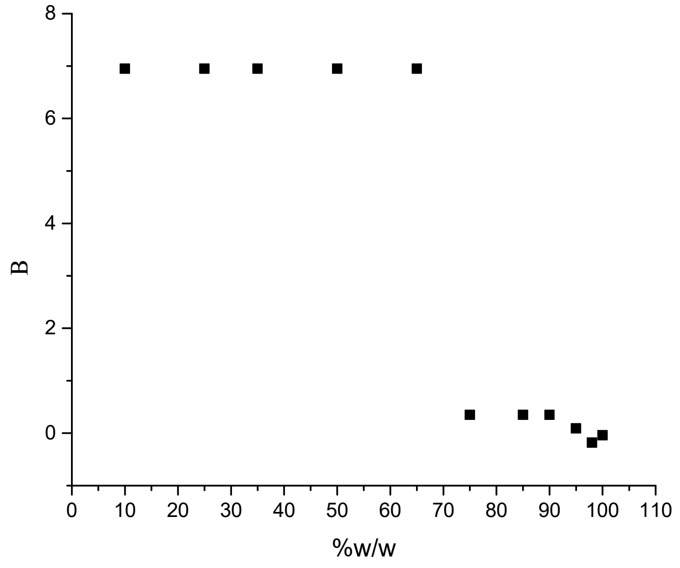

Figure S24. Relationship between β vs. % w/w for CE and its mixtures.

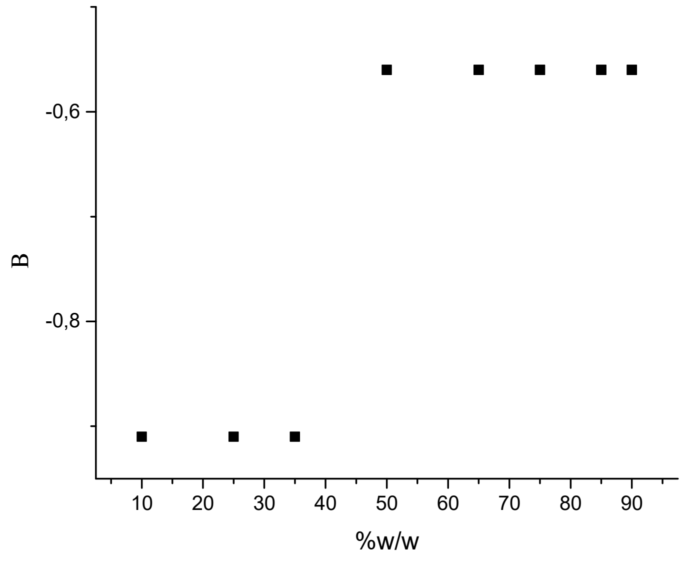

Figure S25. Relationship between ET30 vs. % w/w for CU and its mixtures.

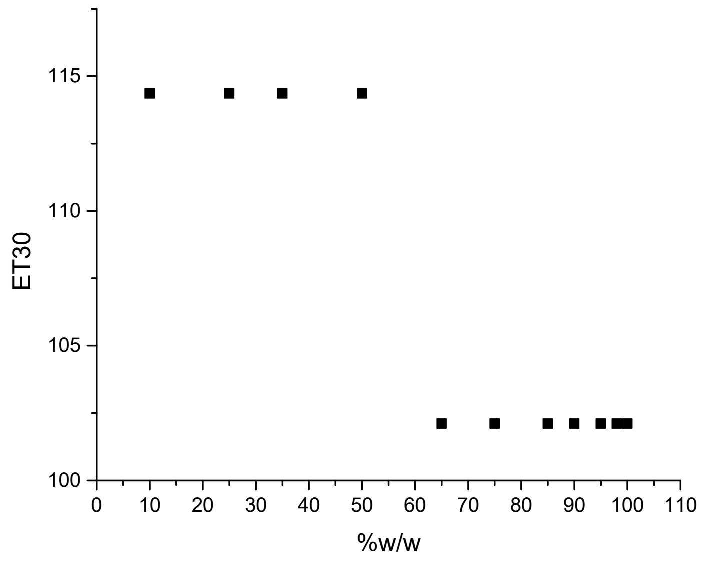

Figure S26. Relationship between ET30 vs. % w/w for CG and its mixtures.

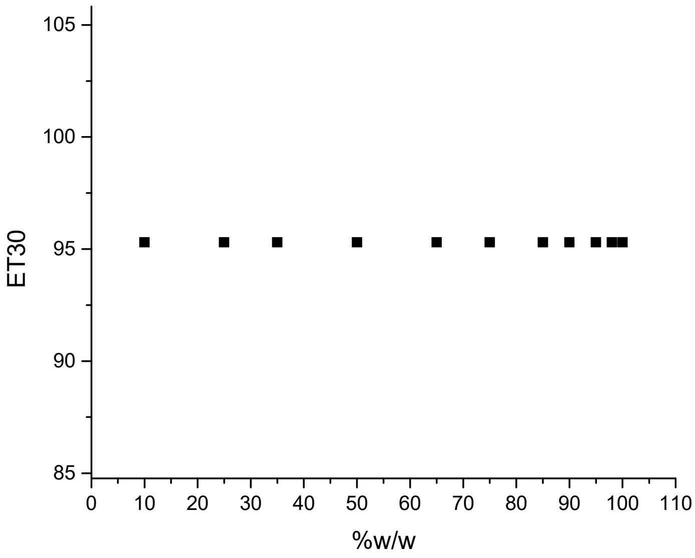

Figure S27. Relationship between ET30 vs. % w/w for CE and its mixtures.

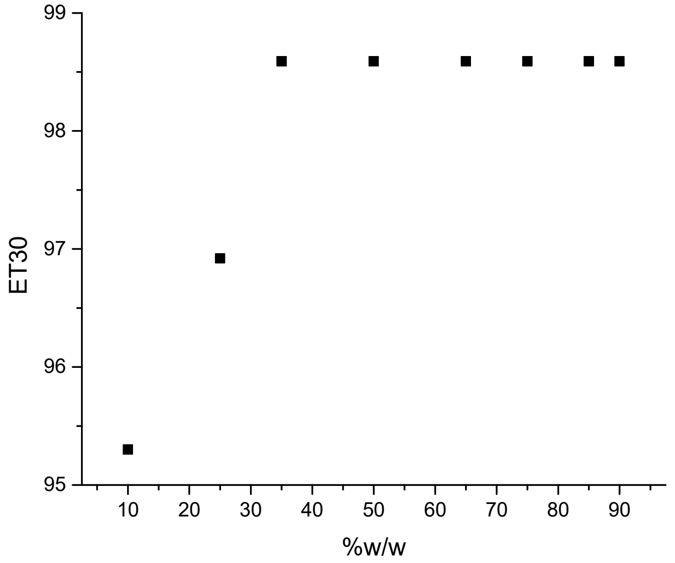

Figure S28. Relationships between conductivity and viscosity in the whole range of % w/w for CU.

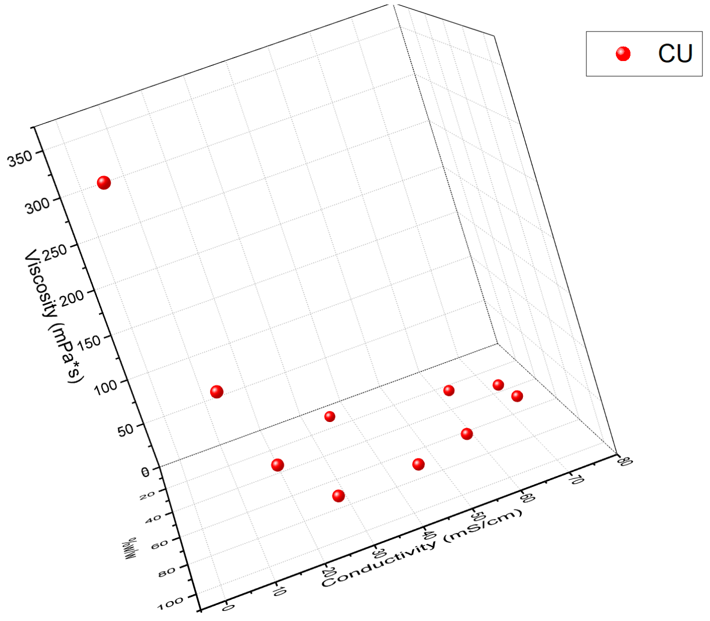

Figure S29. Relationships between conductivity and viscosity in the whole range of % w/w for CG.

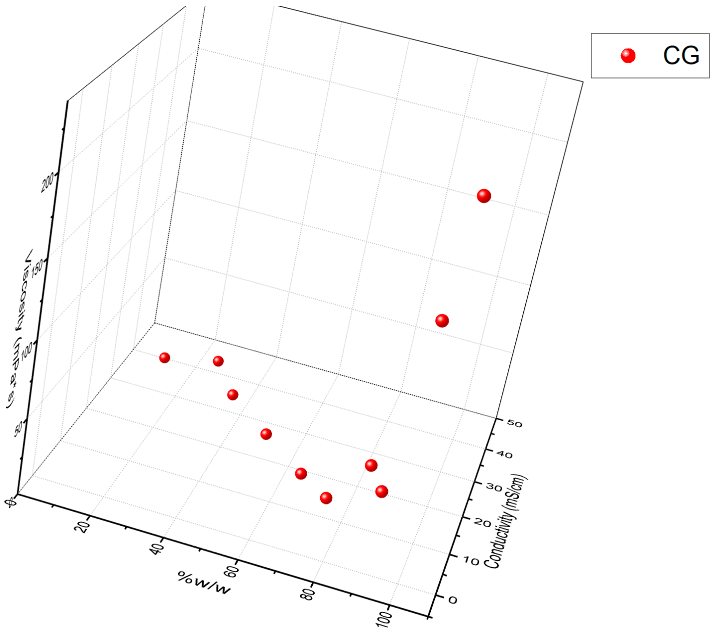

Figure S30. Relationships between conductivity and viscosity in the whole range of % w/w for CE.

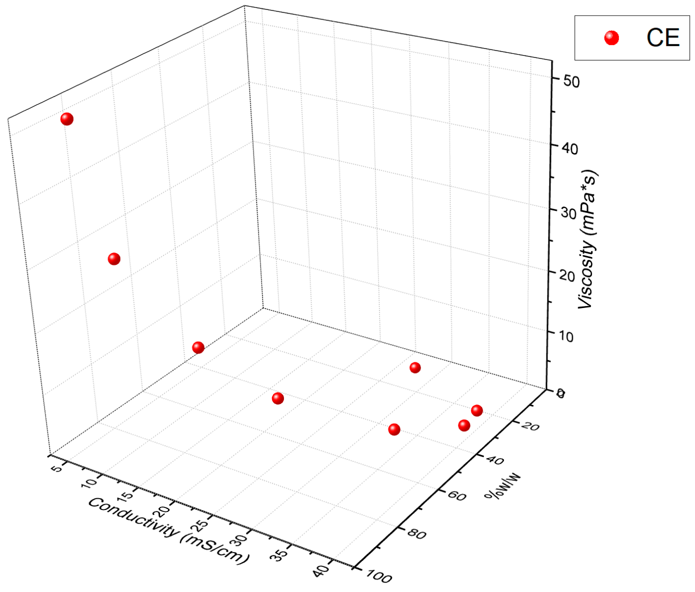

Figure S31. Relationships between conductivity and acidity in the whole range of % w/w for CU.

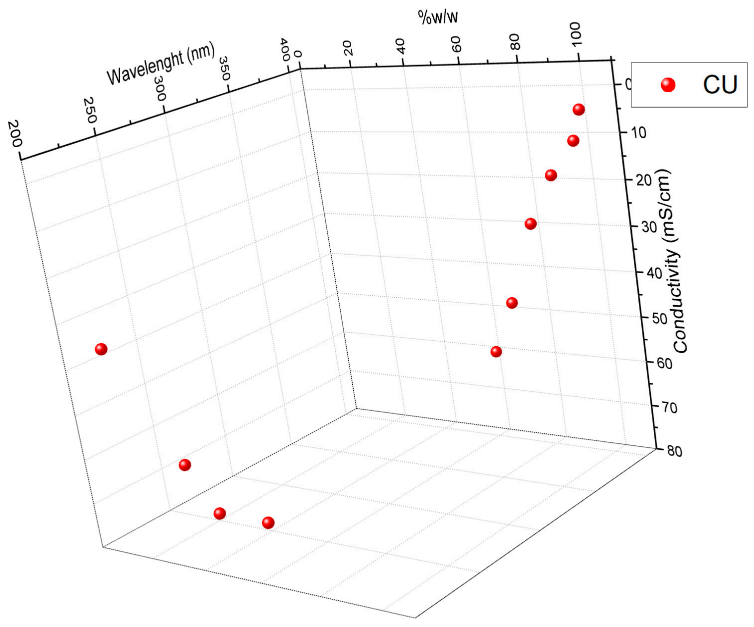

Figure S32. Relationships between conductivity and acidity in the whole range of % w/w for CE.

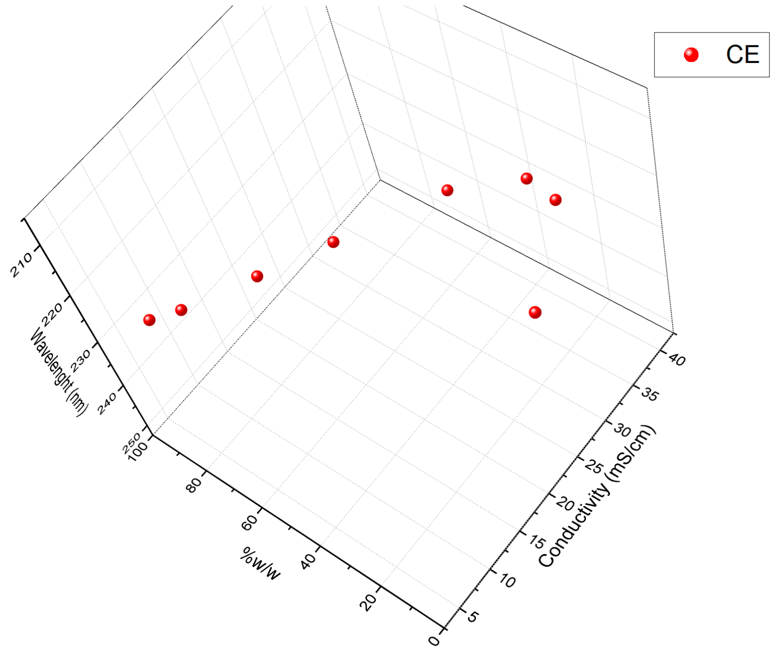

Figure S33. Relationships between conductivity and acidity in the whole range of % w/w for CG.

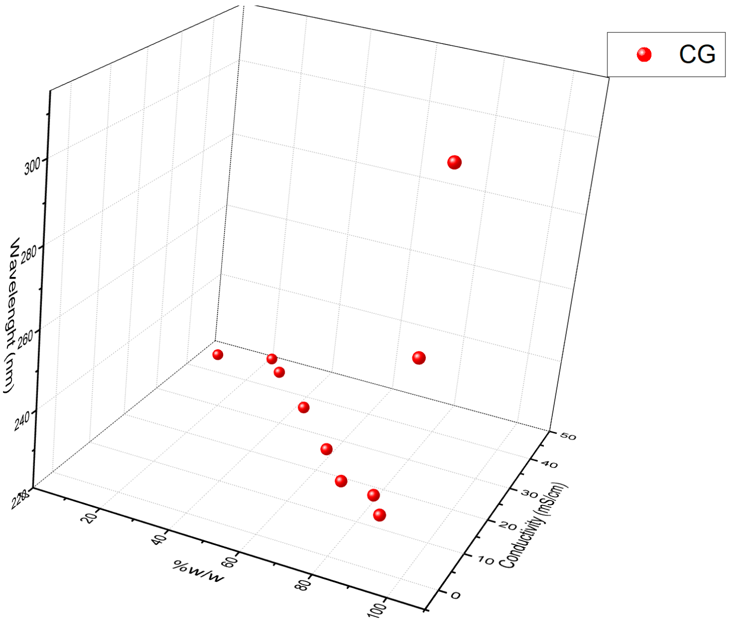

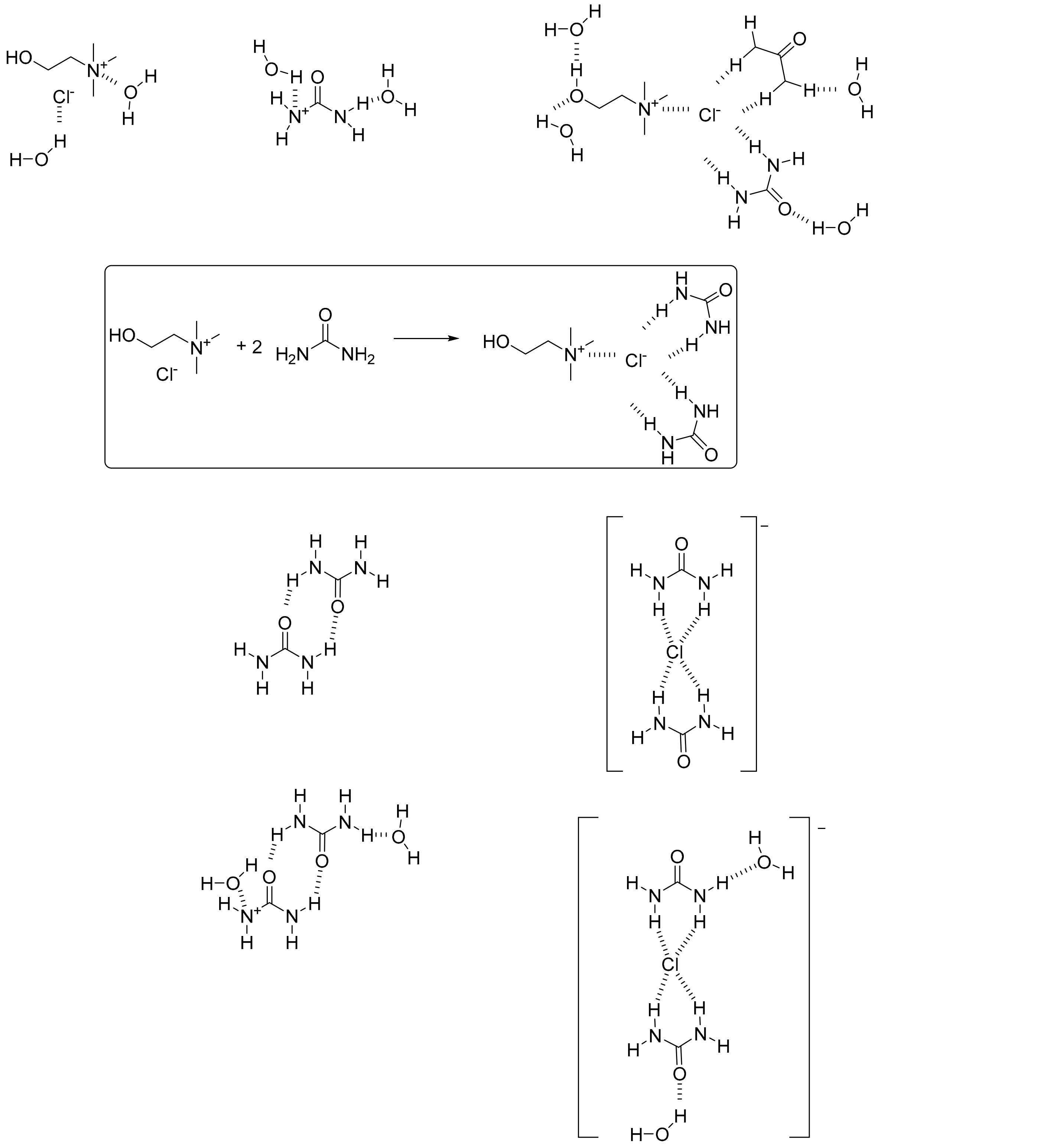

Scheme S2. General picture for CU and some interactions and solvation patterns. This scheme was based on the analysis of: Ashworth, C.R., Matthews, R.P., Welton, T., and Hunt, P.A. ref. [72] Doubly ionic hydrogen bond interactions within the choline chloride-urea deep eutectic solvent. *Phys. Chem. Chem. Phys.* **2016**, 18, 18145-18160. doi: 10.1039/c6cp02815b.
